# Improvement of lipid profile after switching from efavirenz or ritonavir-boosted protease inhibitors to rilpivirine or once-daily integrase inhibitors: results from a large observational cohort study (SCOLTA)

**DOI:** 10.1186/s12879-018-3268-5

**Published:** 2018-07-31

**Authors:** Lucia Taramasso, Paola Tatarelli, Elena Ricci, Giordano Madeddu, Barbara Menzaghi, Nicola Squillace, Giuseppe Vittorio De Socio, Canio Martinelli, Roberto Gulminetti, Paolo Maggi, Giancarlo Orofino, Francesca Vichi, Antonio Di Biagio, Paolo Bonfanti, on behalf of CISAI Study Group, T. Quirino, P. Bonfanti, E. Ricci, C. Bellacosa, P. Maggi, L. Calza, C. Abeli, B. Menzaghi, B. M. Celesia, C. Grosso, A. Stagno, F. Vichi, F. Mazzotta, C. Martinelli, G. Penco, G. Cassola, A. Di Biagio, L. Taramasso, L. A. Nicolini, C. Dentone, C. Molteni, L. Palvarini, A. Scalzini, L. Carenzi, G. Rizzardini, L. Valsecchi, L. Cordier, S. Rusconi, Valeria Colombo, M. Galli, M. Franzetti, G. V. De Socio, A. Sgrelli, E. Mazzotta, G. Parruti, G. Madeddu, P. Bagella, M. S. Mura, R. Libertone, A. Antinori, S. Di Giambenedetto, G. Orofino, M. Guastavigna, P. Caramello

**Affiliations:** 1 Department of Health Sciences (DISSAL), University of Genoa, Genova, Italy; 2 Infectious Diseases Unit, Department of Internal Medicine, Fondazione IRCCS Ca’ Granda Ospedale Maggiore Policlinico, Milan, Italy; 3 Centro Ortopedico di Quadrante, Madonna del Popolo Hospital, Omegna, Italy; 4 Epi2004, Luigi Sacco Hospital, Milan, Italy; 5 Department of Clinical and Experimental Medicine, Unit of Infectious Diseases, University of Sassari, Sassari, Italy; 6 Unit of Infectious Diseases, ASST della Valle Olona, Busto Arsizio Hospital, Busto Arsizio, Italy; 7 Infectious Diseases Clinic, San Gerardo Hospital, University of Milano-Bicocca, Monza, Italy; 8 Infectious Diseases Unit, Santa Maria Hospital, Perugia, Italy; 9 Infectious and Tropical Diseases Unit, Azienda Ospedaliero Universitaria Careggi, Florence, Italy; 10 Infectious Diseases Unit, San Matteo Hospital, Pavia, Italy; 11 Infectious diseases Clinic, Policlinico Hospital, Bari, Italy; 12 Unit of Infectious Diseases, “Divisione A”, Amedeo di Savoia Hospital, Torino, Italy; 13 Infectious Diseases Unit, Santa Maria Annunziata Hospital, Bagno a Ripoli, Florence, Italy; 14 Infectious Diseases Clinic, Policlinico San Martino Hospital, Genoa, Italy; 15 Infectious Diseases Unit, A. Manzoni Hospital, Lecco, Italy; 16 Department of Health and Health Sciences, Policlinico Hospital San Martino, Via Pastore, 1, 16132 Genoa, Italy

**Keywords:** Dyslipidemia, Rilpivirine, Integrase inhibitors, Cholesterol, Framingham risk score

## Abstract

**Background:**

Dyslipidemia represents a significant non-infectious comorbidity among people living with HIV. The aim of this study is to evaluate the impact on lipid profile of switches from an efavirenz (EFV) or protease inhibitor/ritonavir (PI/r)-based regimen to a rilpivirine (RPV) or a once-daily integrase inhibitor-based regimen.

**Methods:**

We analyzed data from SCOLTA prospective database. All patients with HIV-RNA < 50 copies/ml in therapy with two NRTI + EFV or PI/r were included if they switched from EFV to dolutegravir (group EFV-DTG), elvitegravir (EFV-EVG), or RPV (EFV-RPV) and from PI/r to DTG (PI/r-DTG), PI/r to EVG (PI/r-EVG), or PI/r to RPV (PI/r-RPV). Total cholesterol (TC), TC/HDL ratio, LDL-cholesterol (LDL) and triglycerides (TG) were compared at baseline, six months and one year. Comparisons among groups were performed by a general linear model.

**Results:**

Four hundred and ninety patients were enrolled, 24.9% female, mean age 47.3 years (±10.1). According to ART switch, 11.4% were classified in group EFV-DTG, 3.9% in EFV-EVG, 23.9% in EFV-RPV, 17.6% in PI/r-DTG, 17.8% in PI/r-EVG, and 25.5% in PI/r-RPV. After adjusted analysis, TC significantly decreased in all groups but EFV-EVG, TC/HDL in all but EFV-DTG and EFV-EVG, while the reduction of TG was significant only in switches to RPV (EFV-RPV and PI/r-RPV). The one year decrease of TC, TC/HDL, LDL and TG was higher in patients with higher baseline levels of the same variable (*p* < .0001 for all).

**Conclusions:**

In SCOLTA, all switches from PI/r regimens gave advantages on lipid profile, while stopping EFV had consistently favorable lipid effects only if replaced by RPV.

## Background

Cardiovascular disease represents a significant non-infectious comorbidity among people living with HIV in the era of modern antiretroviral therapy (ART) [1]. Dyslipidemia, a well-known cardiovascular risk factor, is frequent and undertreated in this setting [2, 3], where it finds a multi-factorial pathogenesis including host factors, pro-atherogenic HIV action and drug toxicity [4]. Available antiretroviral drugs might favor weight gain, and show variable effects on lipids, with relevant differences both among classes and within the same class [5]. Nucleoside reverse transcriptase inhibitors (NRTIs) do not adversely affect lipids, with tenofovir (TDF) showing a modest lipid-lowering effect [6]. Among non-NRTIs, nevirapine and rilpivirine (RPV) have an overall favorable lipid profile compared to efavirenz (EFV) [7, 8]. Ritonavir-boosted protease inhibitors (PI/r) are globally associated with hypercholesterolemia and hypertriglyceridemia, even if these effects vary with the individual PI/r [9]. Integrase strand transfer inhibitors (INSTIs), especially cobicistat-free ones, have neutral impact on lipids [10, 11]. Nowadays, minimizing drug-related toxicity and improving ART adherence are the main goals of simplification strategies in HIV-suppressed patients. Favorable effects on lipid profile are expected in patients switching to a lipid-friendly, once-daily currently available regimen. However, little is known about the achievement of these expectations in real-life experiences. The aim of this study is to evaluate the impact on lipid profile of a switch from an EFV or PI/r-based regimen to a RPV or a once-daily INSTI-based regimen in a prospective observational cohort of HIV-infected individuals.

## Methods

The SCOLTA (Surveillance Cohort Long-Term Toxicity Antiretrovirals) project is a multicentre observational study started in 2002 that follows HIV-infected people who start a new drug prospectively with the aim of identifying toxicities and adverse events in real life setting [12]. Both ART naïve and experienced patients can be included in the SCOLTA cohort, if they are > 18 years and agree study entry. Demographic, clinical and laboratory data, including total cholesterol (TC), HDL cholesterol (HDL), LDL-cholesterol (LDL) and triglycerides (TG), are prospectively collected in anonymous form in a central database every six months, while adverse events and treatment interruptions are recorded at the moment in which they occur [12]. We performed a query to this prospectively collected database including patients enrolled from its beginning until September 2017 (date of data extraction). We considered all patients in therapy with 2 NRTI associated either to EFV or to PI/r. Patients were enrolled in the study if they had undetectable HIV-RNA (< 50 copies/ml) for at least six months and switched from EFV to dolutegravir (DTG, group EFV-DTG), elvitegravir (EVG, group EFV-EVG), or RPV (group EFV-RPV) or from PI/r to DTG (group PI/r-DTG), EVG (group PI/r-EVG) or RPV (group PI/r-RPV). Patients switched to EVG have been enrolled from July 2012 to December 2016, patients in RPV from February 2013 to December 2016 and patients in DTG from July 2012 to September 2017. Values of TC, HDL, TC/HDL ratio, LDL and TG were compared at baseline, six months (T1) and one year (T2) after the switch. Cardiovascular risk at baseline and T2 was assessed using the Framingham Risk Sore [13], as this algorithm showed a good predictive value in a real-life Italian cohort of HIV infected patients [14]. The study protocol of the SCOLTA Group was approved by local ethical committees and conducted in accordance with the ethical principles stated in the Declaration of Helsinki. Written consent was obtained from all participants.

Patients were described using frequency for categorical variables and mean (standard deviation, SD) or median (interquartile range, IQR) for continuous variables. Comparisons of TC, HDL, TC/HDL, LDL and TG variations in different switch groups were performed at T1 and T2 by GLM least square means procedure, and adjusted for age, sex, years on ART, diabetes, statin use and baseline levels of each variable at multivariable analysis. The analyses among groups were adjusted for multiple comparisons between groups (Sidak correction). Differences were considered significant for *p* values < 0.05.

## Results

During the study period 490 patients satisfied the inclusion criteria and were enrolled. In this population, 122 patients were female (24.9%), the mean age was 47.3 years (±10.1) and mean CD4+ T-cell count 653 (±345) cells/μl. Two-hundred-fifty-four patients (51.8%) were in CDC stage A, 142 (29.0%) in B, and 94 (19.2%) in C. According to the type of ART switch, 56 (11.4%) patients were classified as group EFV-DTG, 19 (3.9%) EFV-EVG, 117 (23.9%) EFV-RPV, 86 (17.6%) PI/r-DTG, 87 (17.8%) PI/r-EVG, and 125 (25.5%) PI/r-RPV. Median cumulative time on ART, expressed in years, was 12.2 (IQR 8.2–17.1) in EFV-DTG, 5.9 (4.7–11.0) in EVF-EVG, 6.0 (3.0–10.8) in EFV-RPV, 10.4 (5.0–17.1) in PI/r-DTG, 8.1 (2.1–16.7) in PI/r-EVG and 4.6 (2.0–11.4) in PI/r-RPV. At baseline, diabetes was recorded for 23 patients (4.7%), equally distributed among study groups (*p* = .1); 47 (9.6%) patients were in therapy with statin and 5 (1.0%) with fibrates. Baseline values of the lipid variables and Framingham risk score are shown in Table 1. During the follow-up, 12 (2.4%) patients started a new treatment with statin, while 6 (1.2%) stopped the statin they were already taking. No patients started or stopped fibrate use during the study.

**Table 1:** Baseline levels of total cholesterol (TC), HDL cholesterol (HDL), LDL cholesterol (LDL), TC/HDL ratio, triglycerides (TG) and Framingham score (FRS) across groups. Variable are expressed as means (±SD) or medians (Q1-Q3)

Variable	Switches from 2NRTI + EFV to			Switches from 2NRTI + PI/r to		
	DTG	EVG	RPV	DTG	EVG	RPV
TC (mg/dl)	198.5 (41.6)	207.4 (40.7)	196.5 (38.0)	209.9 (41.0)	203.7 (43.9)	189.2 (47.1)
HDL (mg/dl)	53.5 (16.7)	49.3 (14.4)	48.8 (13.8)	48.8 (14.7)	43.5 (12.3)	48.8 (21.0)
TC/HDL (mg/dl)	3.93 (1.1)	4.4 (1.1)	4.3 (1.4)	4.65 (1.6)	4.98 (1.6)	4.2 (1.3)
LDL (mg/dl)	117.0 (39.4)	128.2 (32.6)	120.3 (35.5)	126.0 (33.7)	123.5 (38.2)	111.1 (39.5)
TG (mg/dl)	114 (91.5–153.5)	126.5 (106–171)	109 (77–160)	142 (84–215)	150 (112–204)	117 (85–189)
FRS (%)	12.2 (6.7–25.3)	6.7 (3.9–9.4)	7.9 (4.7–13.2)	9.4 (4.5–21.6)	9.4 (4.5–15.6)	7.3 (3.3–13.2)

*NRTI* nucleoside reverse transcriptase inhibitors, *EFV* efavirenz, *PI/r* ritonavir-boosted protease inhibitors, *DTG* dolutegravir, *EVG* elvitegravir, *RPV* rilpivirine

**Fig. 1:**
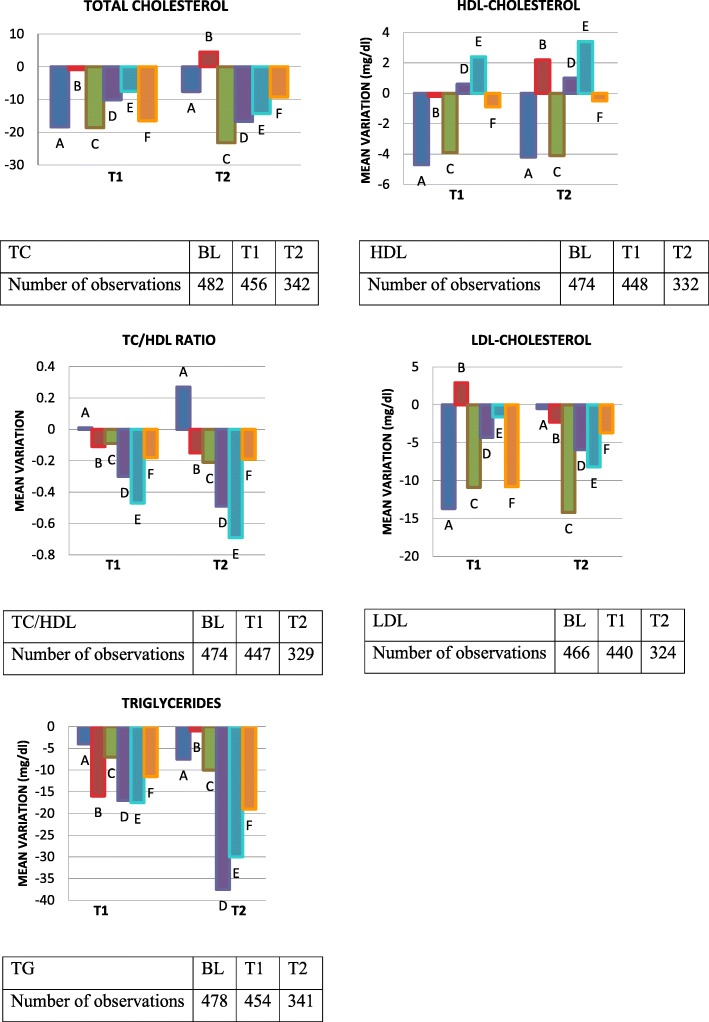
Mean variations of total cholesterol (TC), HDL cholesterol (HDL), TC/HDL ratio, LDL cholesterol (LDL) and median variation of triglycerides (TG) from baseline (BL), to 6 and 12 months of follow up (T1 and T2, respectively) for each switch group (A-F). Tables below each graph show the number of observations at each time-point. Only patients with observations at T1 or T1/T2 are considered in the graph. A: switch from 2 nucleoside reverse transcriptase inhibitors (NRTI) + efavirenz to 2NRTI + dolutegrvir; B: switch from 2NRTI + efavirenz to 2NRTI + elvitegravir; C: switch from 2NRTI + efavirenz to 2NRTI + rilpivirine; D: switch from 2NRTI + protease inhibitors to 2NRTI + dolutegravir; E: switch from 2NRTI + protease inhibitors to 2NRTI + elvitegravir; D: switch from 2NRTI + protease inhibitors to 2NRTI + rilpivirine

Figure 1 shows the trend of the lipid variables across the study, as well as the number of available observations for each one. The frequencies of patients with TC under the standard target level of 190 mg/dl counseled by European AIDS Society guidelines [15] were 43.4% (209/482 patients) at baseline, 59.6% (274/469 patients) at T1 and 60.3% (208/345 patients) at T2. Differences found in TC, HDL, TC/HDL, LDL and TG at T2 were examined after adjustment for age, sex, use of statin, diabetes, years of ART and baseline level of each analyzed variable. Results of multivariable analysis at T2 are summarized in Tables 2 and 3.

**Table 2:** Adjusted mean difference from baseline (mean) and standard error (SE) of total cholesterol (TC), LDL-cholesterol (LDL), total cholesterol/HDL-cholesterol ratio (TC/HDL) and triglycerides (TG) one year after the switch

Switch		TC T2 - TC T0 (mg/dl)		LDL T2 - LDL T0 (mg/dl)		HDL T2 - HDL T0 (mg/dl)		TC/HDL T2 - TC/HDL T0 (mg/dl)		TG T2 - TG T0 (mg/dl)		FRS T2 - FRS T0	
From	To	mean (±SE)	p*	mean (±SE)	p*	mean (±SE)	p*	mean (±SE)	p*	mean (±SE)	p*	mean (±SE)	p§
2 NRTI + EFV	DTG	−15.0 ± 6.5	**.0210**	− 10.9 ± 6.2	.0771	−1.4 ± 1.8	.4222	− 0.21 ± 0.21	.3242	− 19.6 ± 15.2	.1992	0.67 ± 0.71	0.35
	EVG	1.7 ± 8.6	.8401	− 5.9 ± 8.0	.4623	2.8 ± 2.4	.2350	− 0.32 ± 0.27	.2488	27.2 ± 20.5	.1853	0.74 ± 0.92	0.42
	RPV	−31.6 ± 5.3	**<.0001**	−21.0 ± 5.0	**<.0001**	− 2.9 ± 1.4	**.0421**	− 0.52 ± 0.17	.**0021**	−31.5 ± 12.4	**.0116**	0.03 ± 0.38	0.93
2 NRTI + PI/r	DTG	−16.8 ± 5.9	**.0049**	−8.8 ± 5.7	.1247	2.4 ± 1.6	.1335	− 0.60 ± 0.19	**.0015**	−26.1 ± 14.0	.0628	− 0.54 ± 0.60	0.36
	EVG	−16.6 ± 5.8	**.0044**	− 12.0 ± 5.6	.**0312**	3.0 ± 1.6	.0604	− 0.63 ± 0.18	**.0007**	−23.0 ± 16.6	.0926	−0.87 ± 0.52	0.10
	RPV	− 20.7 ± 5.2	**<.0001**	−13.6 ± 5.0	**.0064**	0.00 ± 1.4	.9995	− 0.52 ± 0.17	**.0021**	− 28.2 ± 12.2	**.0216**	−0.25 ± 0.48	0.60

*NRTI* nucleoside reverse transcriptase inhibitors; *EFV* efavirenz, *PI/r* ritonavir-boosted protease inhibitors, *DTG* dolutegravir, *EVG* elvitegravir, *RPV* rilpivirine, *T0* baseline, *T2* one year follow up. Significant *p* values (*p* < 0.05) are indicated by bold font

*Adjustment for age, sex, baseline value of the analyzed variable, time on antiretroviral therapy, diabetes and use of statin

§ Adjustment for baseline value of FRS and time on antiretroviral therapy

**Table 3:** Covariate analysis of total cholesterol (TC), HDL-cholesterol (HDL), TC/HDL ratio, LDL-cholesterol (LDL), tryglicerides (TG) and Framingham risk score (FRS), according to confounding factors used in the multivariable analysis: all confounders and treatment groups were included at the same time

	TC	HDL	TC/HDL	LDL	TG	FRS
Age (b-value, by 1)	0.3 *p* = 0.09	0.0 *P* = 0.92	0.0 *P* = 0.45	0.27 *P* = 0.10	0.1 *P* = 0.89	–
Sex (b-value, F vs M)	6.6 *P* = 0.10	3.4 *P* = 0.007	−0.24 *P* = 0.07	1.7 *P* = 0.65	2.8 *P* = 0.77	–
Diabetes (b-value, Y vs N)	−9.7 *P* = 0.22	0.9 *P* = 0.68	−0.3 *P* = 0.24	−7.4 *P* = 0.34	14.5 *P* = 0.43	–
Statins (b-value, Y vs N)	−10.1 *P* = 0.08	−1.3 *P* = 0.41	−0.0 *P* = 0.66	−7.8 *P* = 0.15	1.1 *P* = 0.93	−0.35 0.62
ART duration (b-value, by 1)	−0.2 *P* = 0.34	−0.1 *P* = 0.37	− 0.0 *P* = 0.86	−0.0 *P* = 0.84	− 0.4 *P* = 0.52	0.02 *P* = 0.58
Baseline value (b-value, by 1)	− 0.4 *P* < 0.0001	−0.3 *P* < 0.0001	− 0.43 *P* < 0.0001	−0.43 *P* < 0.0001	− 0.5 *p* < 0.0001	−0.02 *P* = 0.32

*F* female, *M* male, *Y* yes, *N* no, *ART* antiretroviral therapy

### Total cholesterol

All patients but EFV-EVG group had significant TC decrease at T2. TC trend in EFV-EVG was significantly different to that of EFV-RPV (*p* = .0013), but in all other comparisons the inter-groups differences were not significant. Patients with higher baseline TC experienced the higher TC decrease after the switch (*p* < .0001), while sex, age, years on ART and statin use had no influence.

### HDL-cholesterol

Mean HDL changed differently in different switch groups (*p* = .0002), rising in EFV-EVG, PI/r-DTG and PI/r-EVG, while decreasing in EFV-DTG, EFV-RPV and PI/r-RPV. The HDL variations in each group resulted not significant after adjustment, when compared to baseline levels, with the exception of group EFV-RPV, in which the mean HDL decrease of − 4.1 mg/dl was significant (*p* = .0421). HDL variations were also linked to sex (HDL increased in women as compared to men, *p* = 0.0067) and baseline levels (*p* < .0001), as HDL decrease was more marked in patients with higher baseline level. Age, years on ART and statin use did not influence HDL changes.

### TC/HDL ratio

Mean TC/HLD decreased significantly in all switches from PI/r (group PI/r-DTG, *p* = .0015; group PI/r-EVG, *p* = .0007; group PI/r-RPV, *p* = .0021), while switches from EFV gave significant changes only in RPV group (group EVF-RPV, *p* = .0021). A higher decrease was also found in patients with higher baseline TC/HDL ratio (*p* < .0001), while no differences were found on the basis of sex, statin use, age and different type of switches after multiple comparisons.

### LDL cholesterol

LDL decreased significantly only for switches to RPV (groups EFV-RPV and PI/r-RPV, *p* < .0001 and *p* = 0.0064, respectively) and in the switch from PI/r to EVG (group PI/r-EVG, *p* = .0312). However, the inter-groups differences resulted not significant after adjustment for multiple comparisons, as well as sex, age, years on ART and statin use. Higher baseline LDL levels were instead linked to significant decreases at T2 (*p* < .0001).

### Triglycerides

The mean TG decrease at T2 was statistically significant only for switches to RPV containing regimes (groups EFV-RPV and PI/r-RPV; *p* = .0116 and *p* = .0216, respectively). Multiple comparisons did not find significant inter-groups differences. Patients with higher baseline values were more likely to experience significant TG decreases (*p* < .0001), independently of sex, age and use of statins.

### Framingham risk score

Framingham risk score was calculated for 294 and 187 patients at baseline and T2 (both using the baseline age), respectively. It did not change significantly in any groups. Patients with higher baseline levels experienced the highest risk score reductions, even if not statistically significant and not consistently linear: patients with baseline FRS < 10.0 showed an increase of 0.1, those with intermediate risk a reduction of 1.0, and those at high risk (FRS ≥ 20.0) a reduction of 0.3 (*p* = 0.20). Risk score changes in different groups of switches remained non-significant also after adjustment for baseline Framingham risk score and statin use (Table 2).

### Diabetic patients

With the limit of the low number of diabetic patients included in the study (*n* = 23, 4.7%), we analyzed lipid changes from baseline in this category. We did not find significant differences in those who switched from EFV to any regimens, while diabetic patients who switched from PI/r had significant improvements in lipid profile as compared to non-diabetic patients (TC, − 66.0 vs − 11.0, *p* = 0.0002;; TC/HDL ratio − 1.8 vs − 0.38, *p* < 0.0001; LDL-C -42.2 vs − 4.6, *p* = 0.01).

### TDF containing regimens

Finally, we run a model including also information on TDF use, in both initial and post-switch regimens: the results of the primary analyses were confirmed. Three-hundred and seventy-seven (76.9%) patients were on TDF before and 365 (74.5%) after the switch. In both patients who were initially treated with TDF and those who were not, all the variables showed the same trends described in the general population.

### Treatment failure

Forty-four patients discontinued or changed their therapy before T2, for either clinical reasons or patients’ personal choices. Thirty-two patients experienced at least one detectable HIV-RNA during the follow-up (7 at T1, 18 at T2, 7 both at T1 and T2), with HIV-RNA > 200 copies/ml in 11 cases, of whom 7 had HIV-RNA > 1000 copies/ml.

## Discussion

Here we present the changes of lipid parameters at 12 months of follow-up in a prospective cohort of HIV-infected patients switching from an EFV or PI/r-based regimen to a RPV or a once-daily INSTI-based regimen. We found that replacing PI/r gives advantages on both TC and TC/HDL ratio regardless of the switch group (to DTG, EVG or RPV) and that in case of switch to RPV these advantages extend also on TG and LDL. Also, EFV interruption has a favourable effect on all the studied parameters only if replaced by RPV. These findings suggest that, among the analysed antiretroviral switches, the most favourable lipid impact is obtained by replacing PI/r and EFV with RPV, but significant advantages can also be obtained by replacing PI/r with once-daily INSTI regimens. Other published studies found that switching to RPV-containing regimens leads to an improvement of lipid profile, with some differences among the analysed variables. The SPIRIT study, a randomized clinical trial on safety and efficacy of switching from a PI-based regimen to TDF/emtricitabine (FTC)/RPV in virologically suppressed patients, found significant improvements in TC, LDL, TG and TC/HDL ratio in the immediate switch arm compared with the delayed switch arm [16]. In a multicentre, retrospective study, Pinetti et al. analysed efficacy and safety of treatment simplification to RPV/FTC/TDF in a real-life setting. They found that both TC and TG significantly decreased in patients switching from PI-based regimens, while only TC significantly decreased in patients switching from NNRTI [17]. A significant decrease in TC has also been described in switches from unboosted PIs to RPV [18]. Finally, in a recent study Gagliardini et al. described a significant TC and HDL reduction at one, two and three-year follow-up after switching from EFV/FTC/TDF to RVP/FTC/TDF, while LDL and TG improvement was observed only up to two-year follow-up and no difference was found in TC/HDL ratio over time [19].

In our study, favourable lipids effects were also found in patients switching from boosted PI to DTG or EVG. Two clinical trials on switch from PI to DTG previously reported similar results in both patients with Framingham risk score > 10%, that experienced TC, TC/HDL, LDL and TG significant decrease [20], and in patients with any cardiovascular risk scores, that had TC and TG significantly lower and HDL significantly higher if switched to DTG, compared to patients who continued PIs [21]. In contrast, total cholesterol did not decrease in patients switching from any triple ART to ABC/3TC/DTG in a double-blind randomized trial [22]. Favorable effects on lipids have also been reported in switches to EVG. In the STRATEGY-PI clinical trial patients switching from lopinavir (but not from atazanavir) to TDF/FTC/cobicistat(C)/EVG obtained significant TC decline after 96 weeks [23] and the same was also seen in the SRTATEGY-NNRTI trial in patients switching from EFV to TDF/FTC/C/EVG, although without significant changes in TC/HDL ratio [24]. It has been widely demonstrated that TG/HDL ratio correlates with insulin resistance [25]. Interestingly, in our study only patients switching from PI/r to DTG or EVG (groups D and E) reached the desired outcome of higher HDL and lower TG, that is related to lower insulin resistance [26], while in all the other groups the trend of HDL had the same direction as that of TG. This result suggests that a favorable effect on insulin resistance could be achieved with the withdrawal of the PI/r, a drug class with possible implications in insulin resistance [27]. One of the most interesting finding of our study is that the greater effect on lipids, in switches to once-daily INSTI- or RPV-based regimens, was obtained in patients with higher baseline values of each variable (*p* < .0001 for all) and in diabetic patients as compared to non diabetic ones. This result highlights the importance of ART optimization with low-metabolic impact-regimens especially in patients who have the highest need of lipid control, that are exactly the ones who achieve better outcomes. Current guidelines [15, 28] do not provide indications for type of ART switch in case of dyslipidemia, also if dyslipidemia is mentioned as an adverse event for both EFV, and PI/r and both INSTI and RPV are considered drugs with lower impact on lipids [15, 28]. In Italian guidelines, in the setting of high cardiovascular risk, it is suggested to substitute PI/r with NNRTIs, or with other PIs with lower metabolic impact or with raltegravir [29, 30]. We think that the present study should suggest that, in HIV-infected patients with high cardiovascular risk and who are treated with PI/r or EFV, a switch to RPV or INSTI should be considered. However, ART switch alone is not enough to significantly change the risk of cardiovascular event in HIV-infected patients, as demonstrated by the lack of significant change of the Framingham risk score across time in our series. Although our results encourage switching ART in patients with high cholesterol or triglycerides levels, lifestyle interventions and lipid lowering agents prescription remain keystones for the prevention of cardiovascular disease, and cannot be fully substituted by a switch strategy.

Finally, a low number of virological failures is observed in this study, pointing out that a careful evaluation of patients’ characteristics, historical HIV genotype and drug-drug interactions should always have the priority before planning any therapeutic modifications.

The present study has some limitations. Despite the prospective data collection, the cohorts of patients with different antiretroviral therapies have been enrolled at different times and the study was purely observational. Thus, it is possible that the choice of ART regimen was guided by different patients’ characteristics that may have influenced the results of the analysis, and that the reasons that have guided the decision of starting each single regimen in different study groups have played a confounding role in the analysis. For the same reason, we do not have a randomized control group and cannot demonstrate the causality between ART switch and the measured outcomes. Moreover, lifestyle interventions during follow up have not been registered, and thus we cannot estimate their impact on the study outcomes.

## Conclusions

In our study, in a large cohort of patients prospectively followed up in a real-life context, we found significant improvements of TC and TC/HDL levels in switches from PI/r to the modern once daily INSTI-based therapies and also on LDL in the switch from PI/r to EVG.

Both switches from PI/r and EFV-based therapies to RPV gave advantages in terms of TC, TC/HDL and also TG and LDL values.

## Abbreviations

ART: Antiretroviral therapy
DTG: Dolutegravir
EFV: Efavirenz
EVG: Elvitegravir
FRS: Framingham Risk Sore
HDL: High-density lipoprotein cholesterol
INSTIs: Integrase strand transfer inhibitors
IQR: Interquartile range
LDL: Low-density lipoprotein cholesterol
NRTIs: Nucleoside reverse transcriptase inhibitors
PI/r: Ritonavir-boosted protease inhibitors
RPV: Rilpivirine
SCOLTA: Surveillance Cohort Long-Term Toxicity Antiretrovirals
SD: Standard deviation
T0: Baseline
T1: Six month follow up
T2: One year follow up
TC: Total cholesterol
TDF: Tenofovir
TG: Triglycerides
